# Traction table versus double reverse traction repositor in the treatment of femoral shaft fractures

**DOI:** 10.1038/s41598-018-24317-y

**Published:** 2018-04-13

**Authors:** Ruipeng Zhang, Yingchao Yin, Shilun Li, Lin Jin, Zhiyong Hou, Yingze Zhang

**Affiliations:** Department of Orthopaedic Surgery, Third Hospital of Hebei Medical University, Hebei, China

## Abstract

A novel reduction technique of intramedullary nailing (IMN) for femoral shaft fractures was introduced, and in this study, its therapeutic effect was compared with patients treated with the traditional traction table. From November 2012 to August 2015, the patients with femoral shaft fractures fixed with anterograde IMN were reviewed. Seventy-four patients treated with the traction table and forty-eight patients treated with the double reverse traction repositor (DRTR) met the inclusion criteria of this study. The surgical time, blood loss, open reduction rate and complications were reviewed in this study. The fracture healing was assessed by the radiographs conducted at each follow-up. The functional outcome (hip and knee flexion, Harris Hip Score, and Lysholm knee score) was evaluated at the final follow-up. Average surgical time, blood loss, hip and knee flexion, and Harris Hip Score showed no difference (*P* > 0.05) between the two groups. However, the DRTR was superior to the traction table in fracture healing, Lysholm knee score, open reduction and complications rate (*P* < 0.05). Thus, we concluded that minimally invasive treatment of femoral shaft fractures could be obtained with the DRTR.

## Introduction

Femoral shaft fractures, mostly caused by high-energy trauma, are not uncommon clinically^1,2^. Intramedullary nailing (IMN) and plating are two fixation methods that are usually applied to manage femoral shaft fractures^3,4^. Compared with the plate fixation, better biomechanical effect of femoral shaft fractures could be obtained by IMN^4–7^. Thus, closed reduction and IMN fixation have gradually evolved as the gold-standard technique in the treatment of femoral shaft fractures^8^.

**Figure 1:**
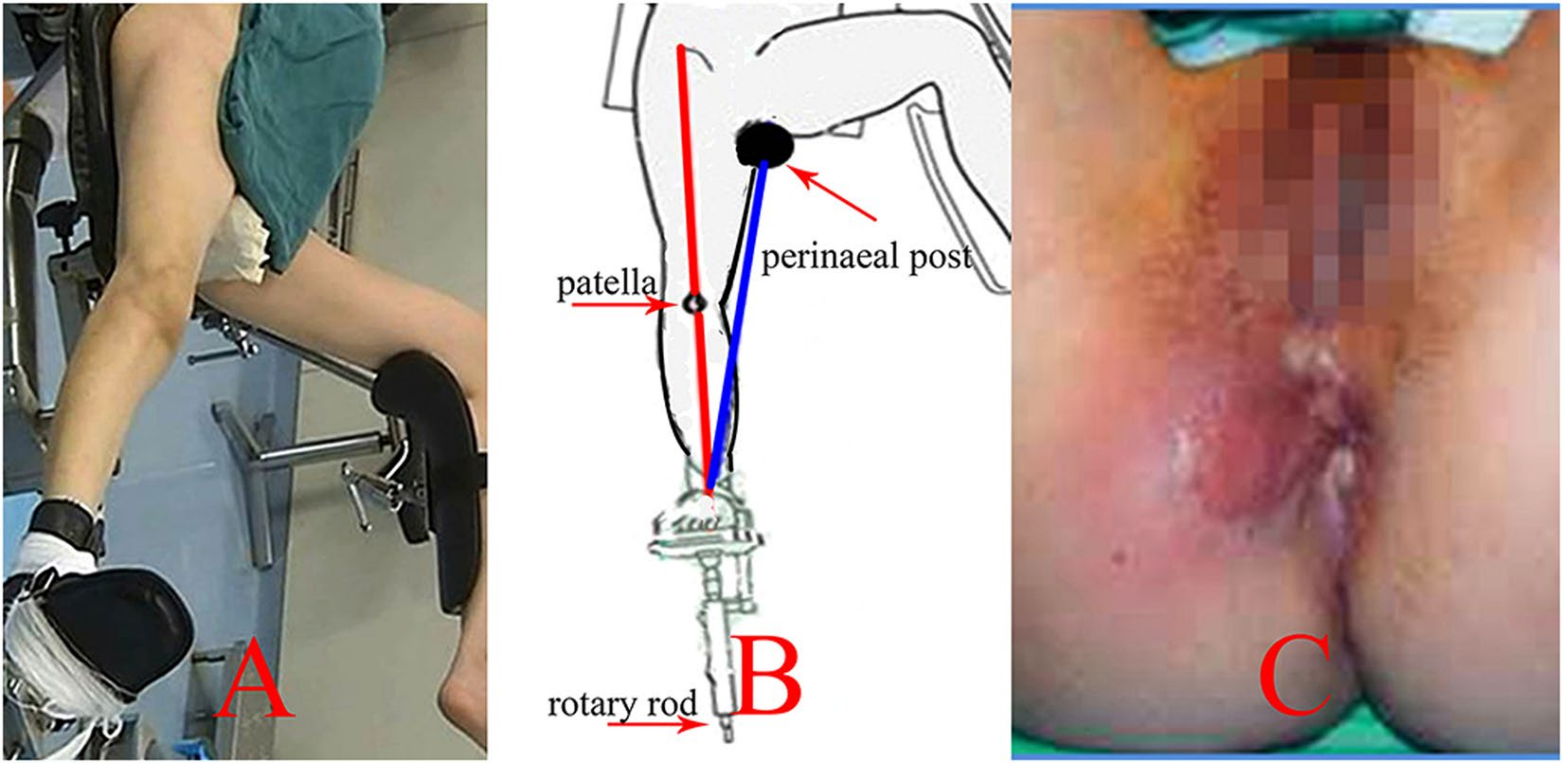
Application of traction table is presented. (**A**) Intraoperative position, (**B**) the tractive axis (blue line) was not parallel with the alignment of lower extremity (red line), (**C**) perineal integument injury after the application of traction table.

As a reduction tool, the traction table could provide continuous and stable tractive strength on the fragments of the lower extremity (Fig. 1A). Thus, it has been widely employed in the IMN surgical procedure for femoral shaft fractures^9,10^. However, the tractive axis is not parallel with the biomechanical line of the lower limb (Fig. 1B). Thus, poor reduction may occur with the traction table^11^. Then, open reduction was indispensable to accomplish the IMN fixation if the guiding wire could not pass through the distal fracture site^12,13^. However, this surgical procedure would seriously damage the blood supply to fragments, which may lead to oligotrophic nonunion^14–16^. Pihlajamaki HK reported that the nonunion rate of femoral shaft fractures treated by the traction table was 12.5%^17^. Additionally, a high incidence of complications, including neurologic and soft-tissue injuries (Fig. 1C), may be accompanied by application of the traction table^1,18,19^.

To resolve these problems, the authors designed a novel reduction tool called the double reverse traction repositor (DRTR). The first purpose of this study was to introduce the novel reduction technique of femoral shaft fractures with the DRTR. The second purpose was to compare the therapeutic results of two different reduction techniques (the traction table and the DRTR).

## Materials and Methods

### Ethical statement

Informed consent was obtained from all the patients and all work was performed in accordance with the guidelines of the institutional internal review board of the participating institution. The experimental protocols were approved by the institutional review board of Hebei Medical University Third Hospital, and the registered number is K2015-001-12.

### Grouping of the patients

A retrospective study to compare the therapeutic effect of femoral shaft fractures treated with different intraoperative reduction tools was conducted in this paper. Patients of femoral shaft fractures fixed with anterograde IMN from November 2012 to August 2015 were reviewed. The inclusion criteria were as follows: >18 years old, unilateral femoral shaft fracture, and longer than 12 months follow-up time. The exclusion criteria were as follows: open fractures, pathologic fractures, fractures in other areas, other risk factors affecting the bone healing (smoking, osteoporosis, metabolic diseases) and non-completion of the one-year follow-up. Overall, 74 patients treated with the traction table (group A) and 48 patients treated with the DRTR (group B) met the inclusion criteria of this study. The fracture types were categorized according to the AO/OTA classification. There were no statistical differences in demographic data between the two groups (Table 1).

**Table 1:** The demographic data of two groups.

Characteristics	Group A	Group B	*P* values
Age (year)	41.05 ± 11.47	39.85 ± 9.88	0.575
Gender M/F	43/31	29/19	0.800
AO Classification (32 A/32B/32 C)	17/38/19	10/25/13	0.958
Time to surgery (day)	5.01 ± 1.67	5.48 ± 2.08	0.212
Follow-up (month)	22.95 (range 13–36)	23.13 (range 12–36)	0.790

### Preoperative examination and preparation

Femoral supracondylar or tibial tubercle traction was conducted in the affected lower limb as soon as possible to decrease the difficulty of intraoperative reduction. Heparin was applied to reduce the incidence of venous thrombosis in this study. A normatively examination, including blood coagulation state and electrocardiogram, was performed to lower the incidence of adverse events intraoperatively. Surgical procedures were conducted when there was no operative contraindication. Thus, the mean time from injury to surgery was more than 5 days in this study (Table 1).

### Surgical techniques

Surgical procedures were carried out by the same team, and the surgical processes of the two groups are described as follows.

For the patients of group A, the unaffected lower extremity was abducted to gain a clear view through C-arm fluoroscope with supine position (Fig. 1A). The radiolucent perineal post and foot piece served as the two traction fulcrums of the surgical extremity (Fig. 1B). A 6-cm longitudinal incision was performed in the area of the greater trochanter to insert the guiding wire, whose entry point was located in the tip of the greater trochanter. The traction strength could be changed by regulating the rotary rod. Adducting, abducting, internal and external rotation of the distal fragment could be obtained by adjusting the position of the foot piece. Rotating of the “gold finger” was performed to achieve insertion of the guiding wire. However, open reduction was essential if the guide wire could not pass through the medullary cavity of distal fragments because of poor reduction or callus blocking. Reaming was routinely performed to insert a nail with a sufficiently large diameter; thus, a relatively stable fixation effect could be obtained. However, extensive reaming should not be done to avoid iatrogenic fracture. Then, the intramedullary nail and locking screws could be placed with the help of radiograph. Static locking was preferred because it could provide adequate stability for fracture sites. However, it was difficult to accomplish the placement of the distal locking screws with the aiming equipment because of the specific anatomy (anterior arch) of the femur. Then, screws had to be inserted with the guidance of continuous exposure of X-ray projection. Dynamic locking was easier because there was a larger space for inserting the screw in the dynamic hole. Thus, distal dynamic locking was also employed in this study to minimize the difficulty of surgery and radiograph exposure.

**Figure 2:**
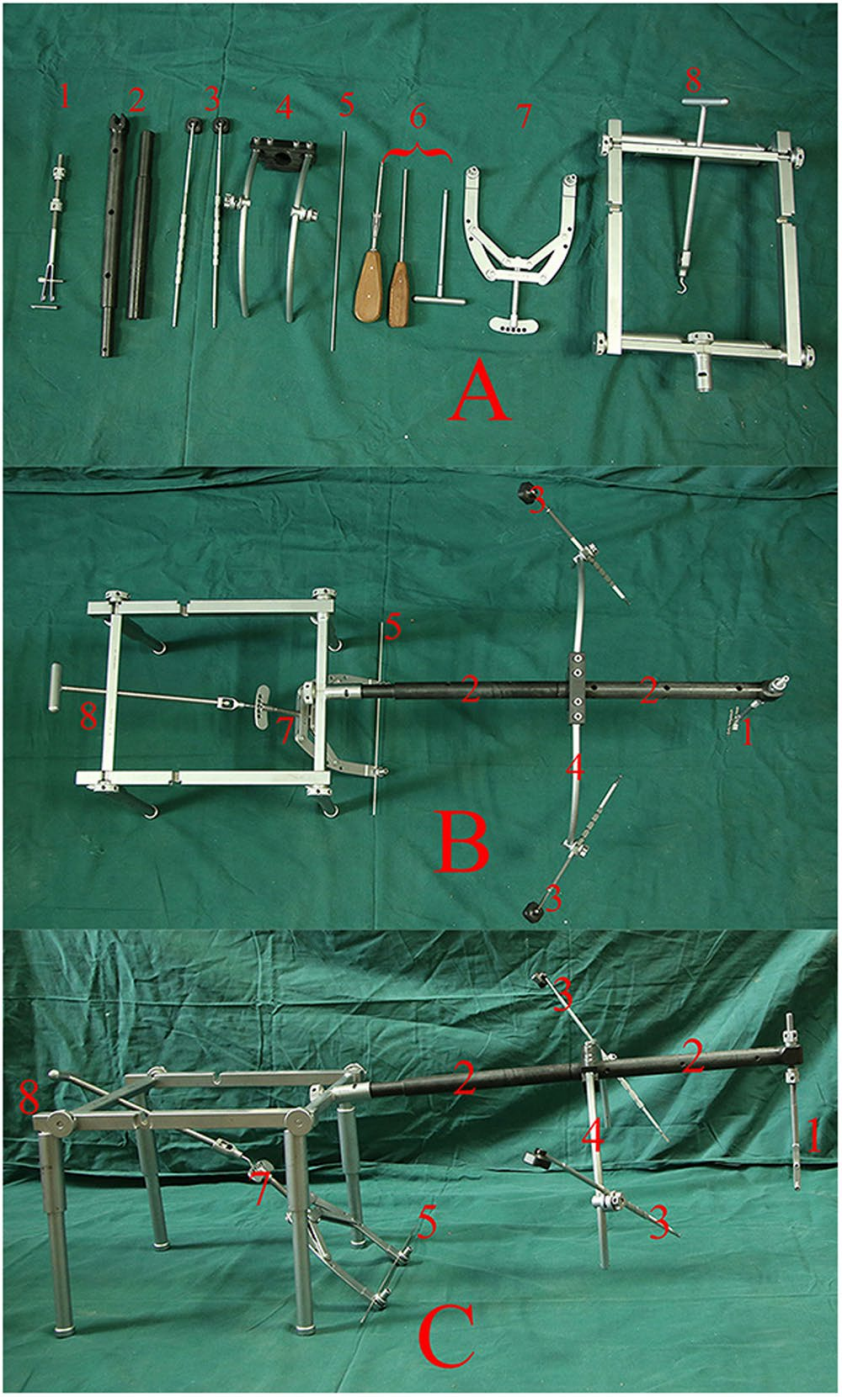
Components, overlooking view and lateral view of DRTR are presented in (**A**–**C**) respectively. (1), Proximal scaffold and 5 mm cortical screw, (2), connecting rod, (3), pushing bars, (4), auxiliary semicircular frame, (5), 2.5 mm Kirschner wire, (6), screwdrivers, (7), traction bow, (8), distal scaffold.

**Figure 3:**
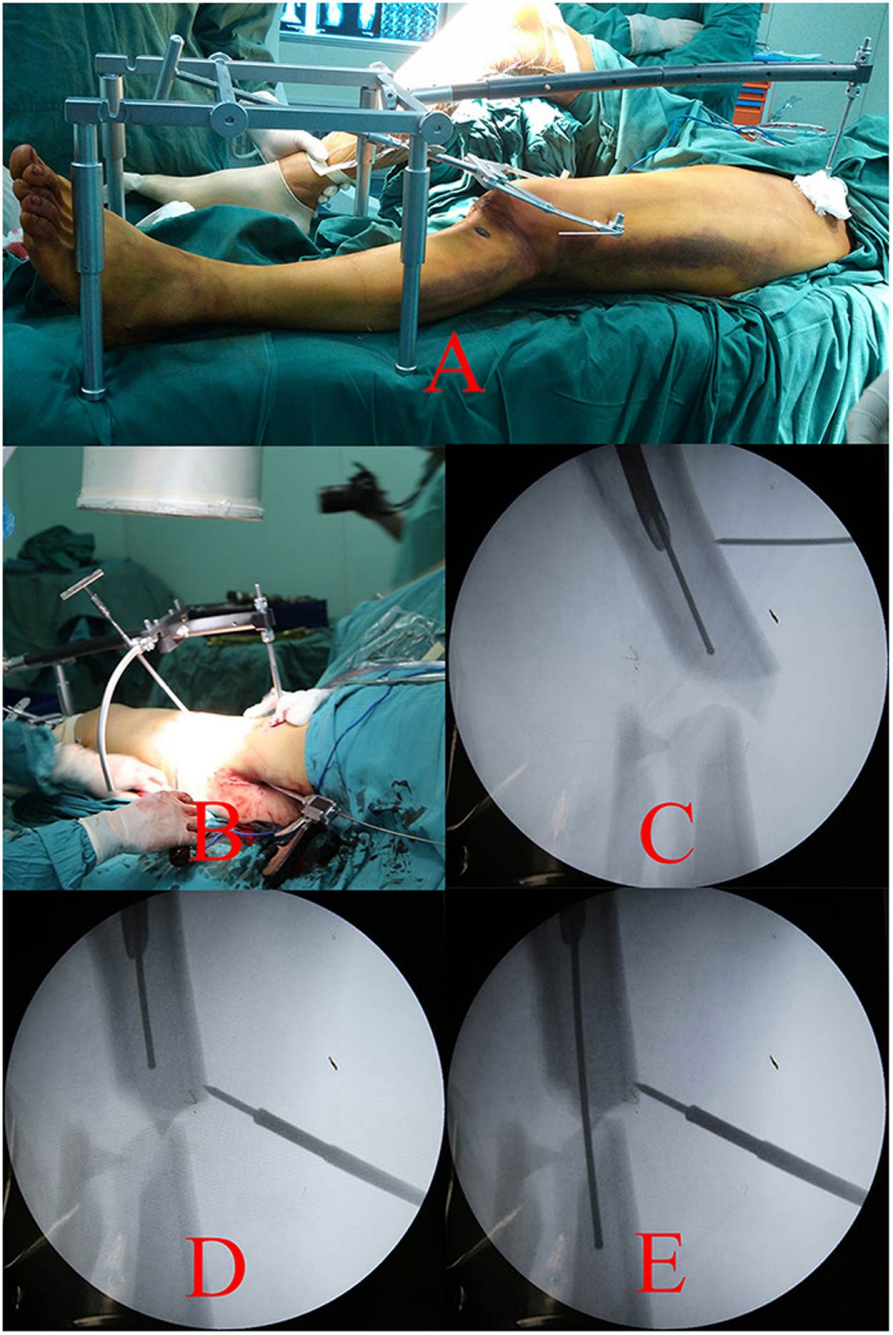
The reduction process of femoral shaft fracture with DRTR was presented. (**A**) Intraoperative view after the DRTR was connected, (**B**–**E**) the inserting of guiding wire was accomplished for the patients with lateral displacement.

The components, overlooking and lateral views of DRTR are presented in Fig. 2. The supine position with a soft cushion under the buttock was recommended during the surgical procedures. The unaffected lower extremity was slightly abducted to obtain clearance of the C-arm fluoroscope (Fig. 3A). A 3-cm incision was performed in the area of the ipsilateral anterior superior iliac spine (ASIS) to fix the proximal pin of the DRTR. Traction with tension via the femoral condyle or the tibial tubercle was conducted using a 2.5-mm Kirschner wire and traction bow. The traction bow was connected to the distal pin with a rotatable hook. Then, the proximal and distal pins could be linked with the connecting rod. Once all parts of the DRTR had been connected, the tractive force could be generated by rotating the distal revolving bar of the DRTR (Fig. 3A). A 6-cm incision in the area of the greater trochanter was performed when the length of the lower limb was restored. Adjusting of the “gold finger” could also be conducted to achieve the insertion of the guiding wire. Moreover, the auxiliary semicircular frame with a pushing bar, as a semi-open technique, was added if there was serious lateral displacement (Fig. 3B–E). Rotational deformities could be reduced by rotating the traction bow. For most cases, the fragments could be reduced with the DRTR. However, open reduction had to be done if the guide wire failed to insert into the medullary cavity of the distal femur. Finally, reaming and IMN fixation were conducted as group A. An application of the DRTR for femoral shaft fracture is presented in the uploaded video.

### Postoperative management

Identical postoperative therapeutic protocols were performed between the two groups. Twenty-four hour antibiotic treatment was routinely conducted to prevent wound infection. Active joint exercise in bed should be conducted to prevent ankylosis postoperatively. Follow-up was performed at one, two, and three months postoperatively and every three months thereafter. The orthopedic-related conditions did not change greatly in the three years postoperatively, thus, the follow-up was finished 36 months later in this study. Patients were encouraged to conduct partial weight-bearing exercise gradually with a walking stick after the surgical wound was healed. Full weight-bearing training should begin when the fracture line disappeared, in anteroposterior and lateral projections at the follow-up.

### Relative clinical data

The patients’ surgical time, open reduction rate, blood loss and complications were reviewed. The surgical time was counted from the success of the anesthesia to the ending of suture, which included the duration of the application of the respective reduction technique. The blood loss was the sum of the blood in the suction bottles and gauzes. Radiograph of the femur was performed at each follow-up to assess the fracture healing. The nonunion was defined as the fracture line that had not completely disappeared within 9 months and had not shown further progression to healing over 3 consecutive months^20^. The Harris Hip Score (HHS), Lysholm knee function score, and hip and knee flexion were assessed at the final follow-up^21,22^.

### Statistical analysis

The statistical data in this study were processed by SPSS (version 23.0; SPSS, Chicago, IL), and a value of *P* < 0.05 was considered statistically different.

## Results

### Surgical comparison

Mean surgical time was 76.89 ± 14.80 min in group A and 82.28 ± 19.78 min in group B (*P* = 0.224). Average blood loss was determined at a mean of 158 ± 50 ml in group A and at a mean of 167 ± 68 ml in group B (*P* = 0.715). Open reduction was conducted in 15 patients of group A and 2 patients of group B (*P* = 0.012) (Table 2).

**Table 2:** Surgical and prognostic comparison of two groups.

Characteristics	Group A	Group B	*P* values
Mean surgical time (min)	76.89 ± 14.80	82.28 ± 19.78	0.224
Mean blood loss (ml)	158 ± 50	167 ± 68	0.715
Open reduction rate	15/74	2/48	0.012
Nonunion	10/74	1/48	0.031
Hip flexion (°)	107.45 ± 13.52	105.31 ± 9.97	0.290
Knee flexion (°)	135.14 ± 14.64	138.13 ± 8.42	0.347
HHS score	89.15 ± 4.90	87.94 ± 4.57	0.150
Lysholm knee score	76.76 ± 6.81	80.56 ± 4.59	0.002

### Prognostic comparison

No wound infection was observed, and all stitches were removed successfully within two weeks in this study. Nonunion was found in 10 patients of group A and 1 patient in group B (*P* = 0.031). A total of 2 cases of group A were hypertrophic nonunion, and the rest were atrophic nonunion in this study. At the final follow-up, the average hip flexion was 107.45° ± 13.52° in group A and 105.31° ± 9.97° in group B (*P* = 0.290). The knee flexion was 135.14° ± 14.64° in group A and 138.13° ± 8.42° in group B (*P* = 0.347). The mean HHS was 89.15 ± 4.90 in group A and 87.94 ± 4.57 in Group B (*P* = 0.150). The Lysholm knee function scoring was 76.76 ± 6.81 in group A and 80.56 ± 4.59 in group B (*P* = 0.002) (Table 2).

### Comparison of complications

Complications occurred in 13 patients of group A and 2 patients of group B (*P* = 0.028). In group A, pudendal nerve palsy developed in 7 patients, and 2 of them had not recovered at the final follow-up. Soft tissue injury of the instep or the perineal integument (Fig. 1C) was observed in 8 patients, and the symptoms disappeared within 2 months’ conservative treatment. Pudendal nerve palsy and soft tissue injury coexisted in 2 patients in group A. For group B, a lateral femoral cutaneous nerve injury occurred in one patient, and an iliaca fossa hematoma developed in another patient, whose symptoms disappeared after 4 weeks of conservative treatment (Table 3).

**Table 3:** The complications of two groups.

Characteristics	Group A	Group B
Pudendal nerve palsy	7	—
Soft tissue injury	8	—
Lateral femoral cutaneous nerve injury	—	1
Iliaca fossa hematoma	—	1

## Discussion

Our results showed that the DRTR was superior to the traction table in fracture healing, Lysholm knee score, open reduction and complication rate.

IMN has become the gold-standard technique in the fixation of femoral shaft fractures. High incidence of complications related to the knee may be accompanied after retrograding IMN^23–25^. Thus, anterograde IMN fixation was preferred by most orthopedists.

The process of intraoperative traction had to be conducted to accomplish the reduction and IMN fixation of femoral shaft fractures. The traction table, providing continuous and stable tractive strength for the lower extremity, has been widely applied to manage femoral fractures^8,20,26^. In the traction procedure, the perineal post and the foot piece served as the two fulcrums^11,27^. The traction force, stepped over three joints (hip, knee, and ankle), was transmitted to the fracture sites through soft tissues (Fig. 1A), which would greatly attenuate the reduction effect. Thus, considerable force, continuously imposed on the perineum and the ipsilateral instep, had to be implemented to obtain the reduction of fragments^28,29^. Iatrogenic damage of the nerve due to excessive traction force has been reported in the literature^11,28–30^. Unfortunately, the recovery of any neurologic defect was unpredictable, and permanent symptoms, including erectile dysfunction, might accompany this procedure^29^.

Soft tissue injuries, including a hematoma of the perineal integument (Fig. 1C) and the instep may also accompany this procedure, because of the continuous force on relevant areas^19,31^. Coelho RF *et al*. reported that additional surgery for debridement and prolonged hospitalization time was indispensable to eliminate infection of the relative areas^31^. Several techniques, including the application of wider perineal post and avoiding excessive adduction of the ipsilateral limb, have been recommended to prevent soft tissue damage^18,32^. However, they were not the fundamental solution for the current problems, because those procedures may influence the reduction effect to a certain extent^27,32^.

**Figure 4:**
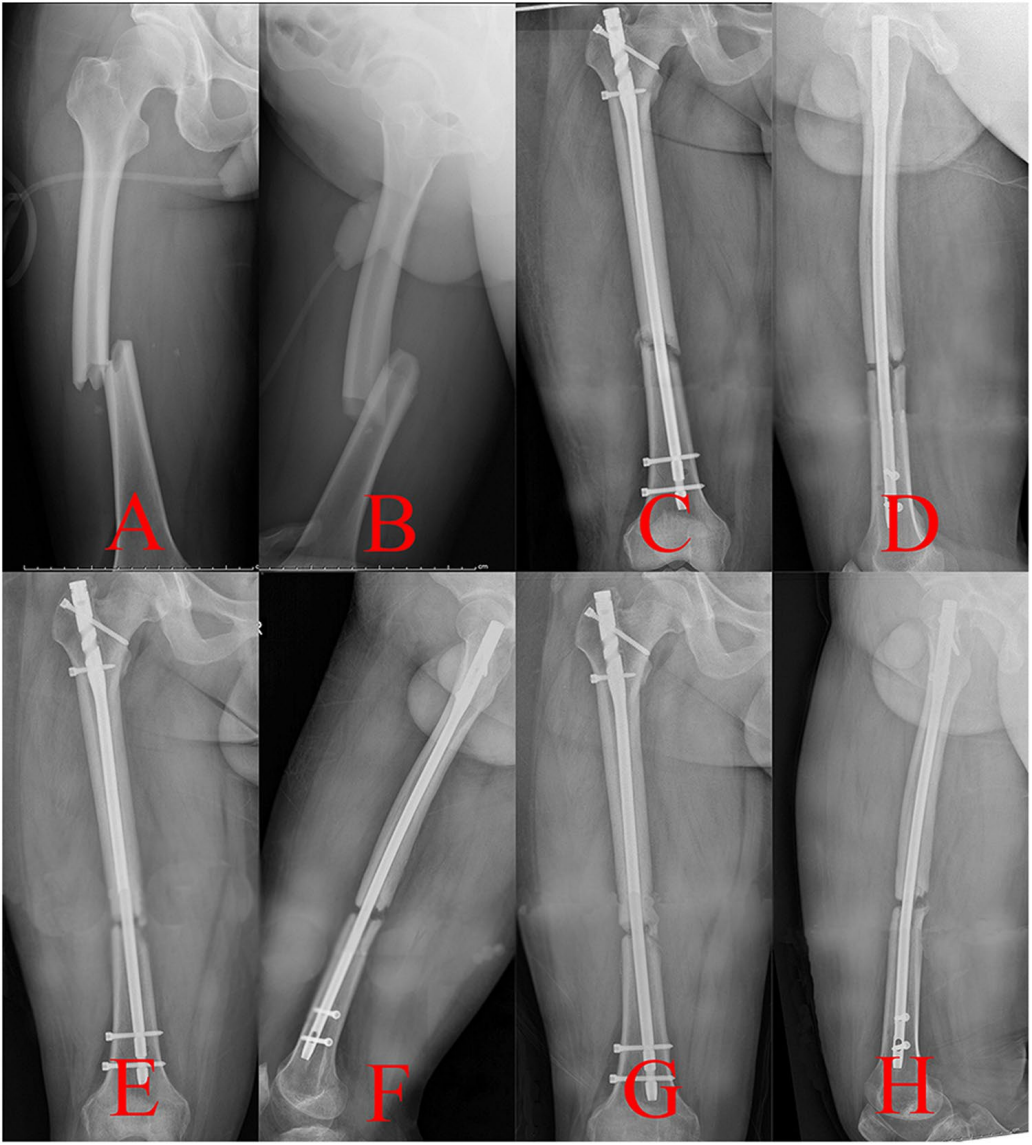
Radiographs of a nonunion case after open reduction in group A were presented. (**A**,**B**) Preoperative anterior-posterior (AP) and lateral views, (**C**,**D**) AP and lateral views a month postoperatively, (**E**,**F**) AP and lateral views 6 months postoperatively, (**G**,**H**) AP and lateral views a year postoperatively.

Nonunion of the femoral shaft fracture was another common complication for the patients treated with the traction table, which could be classified as hypertrophic nonunion caused by the instability of fixation and atrophic nonunion that resulted from biological impairment^33,34^. Second surgical procedures, including exchanging a larger-diameter nailing and bone graft, were indispensable, which would bring great burden to the patients^2,35–37^. The mechanical axis of the lower extremity is stretched straight from the ASIS to the midpoint of the ankle joint^11,38^. However, if the tractive force was not parallel with the mechanical axis of the lower extremity^11^ (Fig. 1B), a poor reduction quality of fracture sites may be obtained. Open reduction may be essential to accomplish an IMN fixation of a femoral shaft fracture. The procedure would seriously damage the blood supply of fragments, which may lead to atrophic nonunion of the fracture (Fig. 4)^15,16^. Thus, a relatively high incidence of atrophic nonunion was observed in this study.

**Figure 5:**
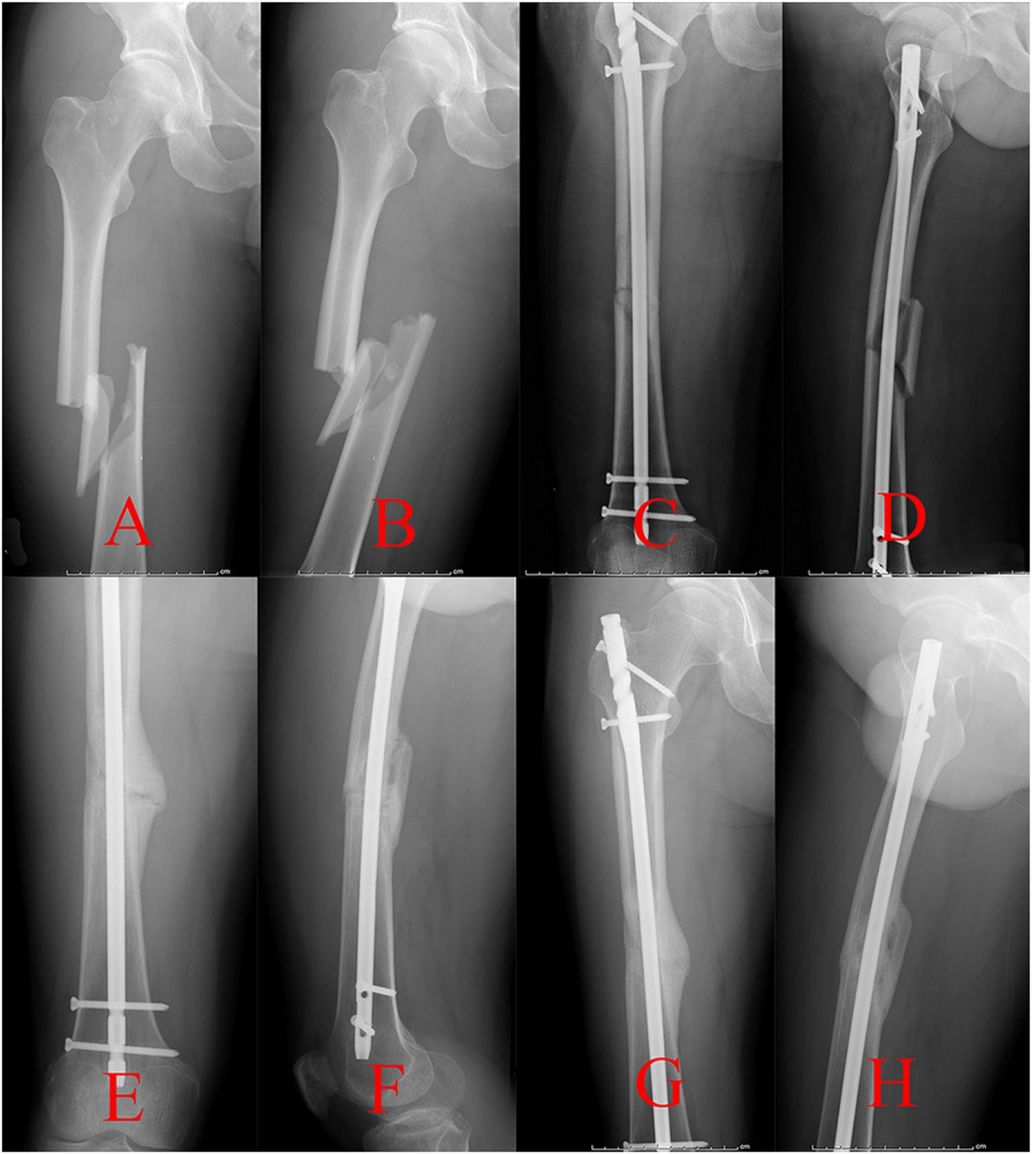
Radiographs of a union case after closed reduction in group B were presented. (**A**,**B**) Preoperative AP and lateral views, (**C**,**D**) AP and lateral views a month postoperatively, (**E**,**F**) AP and lateral views 6 months postoperatively, (**G**,**H**) AP and lateral views a year postoperatively.

The authors designed a novel kind of reduction instrument called the DRTR to resolve the relative problems of the traction table (Fig. 2). Taking a femoral shaft fracture as an example, the two fulcrums of the traction force are located in the ASIS and the femoral condyle (or the tibial tubercle) (Fig. 3A). Thus, the traction alignment was parallel with the mechanical axis of the lower extremity. The traction force of the DRTR, stepped over the hip (and the knee), was transmitted by bone. Thus, compared with the traction table, less tractive force was required to acquire the same reduction effect for the patients treated with the DRTR, which would greatly lower the incidence of soft tissue injuries. Lateral and rotational deformities may be accompanied because the insertions of the femoral antergic muscles are located in different levels. The semi-open technique (auxiliary semicircular frame with a pushing bar) could be employed to reduce the lateral fragments effectively (Fig. 3B–E). Additionally, compared with the open reduction procedure of the traction table, this approach was less invasive. The reduction of the rotational deformity could be gained by rotating the traction bow. Compared with the traction table, a lower open reduction rate could be obtained with the application of the DRTR. Thus, the blood supply of fracture sites was protected, which may be the reason for the higher incidence of union of group B in this study (Fig. 5). Open reduction also had to be performed for the seriously displaced fragments, whose reduction could be achieved by fingers or a ball-spiked pusher in the surgical process. However, further reduction methods, including wire cerclages, were not recommended in this study because the procedure would seriously damage the blood supply to the periosteum, which may have a negative effect on the fracture healing. The mean Lysholm knee function score was higher in group B mainly because there was a relatively lower rate of complications and nonunion for the patients treated with the DRTR.

A 3-cm incision in ASIS was conducted, and relevant complications may be accompanied with the DRTR. Lateral femoral cutaneous nerve injury may occur during the dissection because of its highly variable course and branches; however, the symptoms, such as paralysis and pain at the anterolateral thigh, could disappear after months of conservative treatment^39,40^. Although an extra incision was performed in group B, mean blood loss and surgical time showed no significant difference between the two groups, which may have resulted from the lower rate of open reduction in group B. Additionally, the DRTR is much cheaper than the traction table, which would greatly benefit primary hospitals.

There were some limitations in this study. As a retrospective study, the patients were not randomly divided into two groups. The study may also be limited by the relatively small sample size and short follow-up period, which may not represent the traits of all femoral shaft fractures. More patients should be recruited, and relevant study in this field should be conducted in the future in order to further explore the feasibility of DRTR for femoral shaft fractures.

In conclusion, minimally invasive treatment of femoral shaft fractures could be obtained with the DRTR.

## Electronic supplementary material

Supplementary video
